# The role of kindling mechanism: A validation study of the Hungarian version of the Prediction of Alcohol Withdrawal Severity Scale

**DOI:** 10.1371/journal.pone.0330629

**Published:** 2025-09-02

**Authors:** Otília Bagi, Bettina Kata Kádár, Fanni Fruzsina Farkas, Janka Gajdics, Ildikó Katalin Pribék, Bence András Lázár

**Affiliations:** Addiction Research Group, Department of Psychiatry, University of Szeged, Szeged, Hungary; Belgrade University Faculty of Medicine, SERBIA

## Abstract

**Background:**

Early recognition of the complicated form of alcohol withdrawal syndrome (c-AWS) is critical. The Prediction of Alcohol Withdrawal Severity Scale (PAWSS) was developed for the risk analysis of the development of c-AWS. According to the kindling mechanism, the history of previous c-AWS has a pivotal role in the development of current c-AWS. The aims of this study were to reveal (1) the psychometric characteristics of the PAWSS among patients with alcohol withdrawal syndrome (AWS) and alcohol dependence syndrome (ADS) and (2) the role of kindling mechanism in the development of c-AWS by using the PAWSS.

**Methods:**

This study enrolled 70 inpatients with ADS and AWS. The severity of dependence was measured using the Alcohol Use Disorder Identification Test and the Severity of Alcohol Dependence Questionnaire. Statistical analyses were performed using receiver operating characteristic (ROC) analysis, binary logistic regressions, and for the inter-rater reliability analysis Cohen’s Kappa coefficient was calculated.

**Results:**

ROC analysis showed that > 6 is the optimal cutoff point for the Hungarian version of the PAWSS. In the case of predictive validity, higher PAWSS score (*p* < 0.001) predicted current c-AWS. Furthermore, the history of c-AWS (*p* < 0.001) was a significant variable for current c-AWS. The Cohen’s Kappa coefficient resulted in being 1.

**Conclusions:**

The probability of current c-AWS was 12 times higher among patients with PAWSS scores of 6 or higher. The chance of current c-AWS was almost 7 times higher in the case of history of c-AWS. These findings suggest that the Hungarian version of PAWSS is a valid and reliable clinical tool for assessing the risk of c-AWS, and highlight the importance of the kindling mechanism in the background of c-AWS.

## Introduction

Alcohol withdrawal syndrome (AWS) is one of the most common consequences of alcohol dependence syndrome (ADS) and usually occurs in the first 24 hours after the cessation of alcohol use [1–3]. Approximately 50% of patients with ADS present with AWS during their lifetime [4,5]. Although most AWS cases are mild to moderate, approximately 20% of withdrawal states are complicated with alcohol-related seizure (ARS) and/or delirium tremens (DT), which can be life-threatening [4,5]. Early recognition, diagnosis, optimal follow-up, and the treatment of complicated AWS (c-AWS) are critical for preventing fatal outcomes.

The diagnosis of AWS is a clinical diagnosis based on the Diagnostic and Statistical Manual of Mental Disorders (DSM) and International Classification of Diseases (ICD) diagnostic criteria. Nevertheless, clinical assessment scales, such as the Clinical Institute Withdrawal Assessment Scale for Alcohol, Revised (CIWA-Ar), are important in the early recognition of AWS and the monitoring of changes in symptoms [6–8]. The CIWA-Ar is the most used scale in clinical settings and scientific projects. It has been demonstrated that a decrease in the total CIWA-Ar score is associated with a decrease in withdrawal symptoms, and it is possible to discriminate the efficacy of treatment options [7]. CIWA-Ar has been translated into various languages [8–11], and several guidelines suggest it as a ‘gold-standard’ tool in the management of AWS [12,13]. However, during the last two decades, the use of CIWA-Ar has been suggested to have various limitations [3]. For example, it is important to note that the validation of the original version of CIWA-Ar was performed in a select population of patients with mild to moderate AWS. Furthermore, no vital sign assessment was performed, which would have been important in the risk detection of developing DT. Also, the item related to seizure was dropped off from the original (CIWA) scale. In addition, it has been demonstrated that the decrease in CIWA-Ar scores is independent of the presence of ARS [7]. Therefore, CIWA-Ar might not be the best option for monitoring patients with complicated withdrawal syndrome.

Early recognition of the risk of complicated withdrawal syndrome is crucial, since the severity of AWS is generally determined only when ARS and DT occurs [14]. Our previous findings revealed various risk factors for ARS and DT development [15,16].

The kindling hypothesis has been supported over the past few decades, underlying the development of future episodes of AWS with ARS [16,17]. Furthermore, the central nervous system’s increased excitability resulting from recurrent seizures can lead to the brain’s development of an epileptogenic state, making the history of ARS a significant risk factor for seizures during withdrawal syndrome [18]. Recent studies have suggested that kindling also plays a significant role in the development of DT [16]. Furthermore, in agreement with earlier studies, findings suggest the importance of the kindling mechanism in the development of these conditions [16]. It has also been demonstrated that the history of AWS, ARS, and DT are predictors of future c-AWS episodes [16,18]. Although various studies have suggested the importance of the kindling mechanism in the development of c-AWS, follow-up studies have not yet been published. Moreover, several risk factors have been identified in the development of c-AWS [15,16,18,19]; however, the relationship between the severity of ADS and the development of complicated withdrawal has not yet been evaluated in detail.

Recently, Maldonado and his colleagues developed and validated a new scale, the Prediction of Alcohol Withdrawal Severity Scale (PAWSS) [4,5]. In contrast to other withdrawal scales, including the CIWA-Ar, this tool can detect the risk of developing c-AWS. The 10-item scale focuses on the patients’ ADS history and current state. PAWSS was developed for the prediction of severe AWS and has been validated among patients hospitalized in general internal medicine and surgery departments [4,5]. Although the PAWSS has been validated among a specific population of patients, the severity of alcohol dependence syndrome has not been revealed among these patients, and its sensitivity and specificity have been determined using the CIWA-Ar, the PAWSS has excellent psychometric characteristics and predictive value [4]. Furthermore, the PAWSS contains two items that are related to the kindling mechanism: the history of ARS and DT.

To the best of our knowledge, the PAWSS has not been translated into other languages, and its psychometric characteristics have not been determined in other countries, yet. Therefore, the main goal of this study was to reveal the psychometric characteristics of PAWSS among patients hospitalized primarily with AWS and ADS. Additionally, the other aim of the present work was to reveal the role of kindling mechanism in the development of complicated withdrawal using the PAWSS.

## Methods

### Participants

As a part of a larger study, we recruited a total of 70 inpatients at the Department of Psychiatry, University of Szeged, Hungary between 1 September 2022 and 31 December 2023. Inclusion criteria included any patient with the diagnosis of alcohol dependence syndrome (F10.20) and the principal diagnosis of alcohol withdrawal state (F10.30) based on the 10th revision of the International Classification of Diseases (ICD-10), 18 + years of age, and fixed-schedule regimen with chlordiazepoxide. Patients who had clinically significant somatic- and/or neurological disorders, such as schizophrenia, major depressive disorder with psychotic features, neurocognitive disorders; had any antidepressant or antipsychotic treatment; had any other comorbid addictions were excluded from this study. Before the enrolment, every patient signed a written informed consent form.

The study was conducted in accordance with the Declaration of Helsinki and was approved by the Human Investigation Review Board, University of Szeged (ethical approval number: 81/2022-SZTE RKEB).

### Procedure

The translation process was carried out using the “back translation method” by two psychiatrists with expertise in alcohol dependence treatment and an advanced degree in English. After translating the PAWSS questionnaire into Hungarian, it was reviewed by our research team in terms of face validity, then translated back into English and was compared to the original form. The test battery included the following questionnaires: Prediction of Alcohol Withdrawal Severity Scale (PAWSS), Severity of Alcohol Dependence Questionnaire (SADQ), Alcohol Use Disorders Identification Test (AUDIT) and basic questions about the demographic characteristics (age, sex) of the participants.

### Measurements

#### Prediction of Alcohol Withdrawal Severity Scale (PAWSS).

The PAWSS was designed by Maldonado and his colleagues to identify individuals who are at high risk of developing c-AWS (ARS and DT). This questionnaire helps determine a patient’s probability of severe alcohol withdrawal by evaluating their baseline risk factors before symptoms occur allowing for the implementation of prophylactic treatment. According to the results of the original article, the PAWSS score of 4 or higher predicts complicated withdrawal [4].

#### Severity of Alcohol Dependence Questionnaire (SADQ).

The SADQ is a widely used questionnaire to help determine the level of alcohol dependence in patients diagnosed with alcohol dependence syndrome. The SADQ is self-administered, includes 20 items and focuses on a recent period of heavy drinking from the last 6 months. Regarding the scores, 31 or higher indicates severe alcohol dependence [20].

#### Alcohol Use Disorders Identification Test (AUDIT).

The AUDIT assesses hazardous and harmful alcohol use. It is a brief screening instrument consisting of 10 questions that measures the quantity and frequency of alcohol intake, typical drinking patterns and problems caused by alcohol misuse. Scores of 8 and above are considered to indicate hazardous and harmful alcohol use [21].

### Statistical analysis

Prior to the start of the present study, sample size analysis was conducted using the MedCalc Statistical Software [22]. IBM SPSS 24 [23] was used for statistical analysis and statistical significance was considered if p < 0.05. A Receiver Operating Characteristic (ROC) [24] analysis was conducted to ascertain the optimal threshold value to test the scale’s performance, and the Youden Index [25] was also calculated to measure the effectiveness of the PAWSS. According to Kallner the Youden’s index is better the closer it is to one [26]. The specificity and sensitivity (indicators of a scale’s quality), positive (PPV) and negative (NPV) predictive value (indicators of a tool’s efficiency), and the presence of the type I (false positive, FP) and type II (false negative, FN) errors were calculated to determine the optimal cutoff for the scale. Based on the results, the cutoff point of 6 was used for the Hungarian version of the PAWSS.

For further analyses two groups were created. The first group was the ‘non-c-AWS’ group, which included patients who did not have c-AWS. The second group was the ‘c-AWS’ group, composed of patients who had ARS and/or DT (i.e., c-AWS).

The history of c-AWS variable is a part of the PAWSS. There are two separate questions in the PAWSS which reflect to the history of alcohol-related seizure (ARS) and delirium tremens (DT), which characterise the history of c-AWS. These two questions were used to create the history of c-AWS variable for the analyses.

Regarding the psychometric analysis of the PAWSS, inter-rater reliability was assessed according to Maldonado and his colleagues [4], and in the case of validity, face validity (see Procedure paragraph) and predictive validity were examined. In the predictive validity analysis, the PAWSS score of ≥6 was investigated, whether it predicts current c-AWS with the control of alcohol use severity and demographic variables. Therefore, binary logistic regression analysis [27] was used, the dependent variable was the presence of a current c-AWS, the independent variables were the following: age, sex, the SADQ total score and a PAWSS score of ≥6.

A chi-square test was conducted to estimate the rate of the difference between current c-AWS and the history of c-AWS. In addition, another binary logistic regression was performed to examine the potential variables which predict current c-AWS. The aim of this analysis was to explore the role of the kindling mechanism in the development of c-AWS. The dependent variable was the presence of current c-AWS, the independent variables were age, sex, and the history of a c-AWS.

## Results

### Sample characteristics

In the total sample, 72.9% (n = 51) were male and 27.1% (n = 19) were female. Mean age was 50.04 (SD = 9.86) years. The AUDIT mean score was 28.17 (SD = 6.04) which indicated severe ADS. Furthermore, 35.7% (n = 25) had c-AWS in the past, and 32.9% (n = 23) had c-AWS during the current hospitalization. The mean PAWSS score was 4.93 (SD = 1.41) and 78.6% (n = 55) of the patients had a PAWSS score of 4 or above.

### Psychometric analysis of the PAWSS

**Fig 1:**
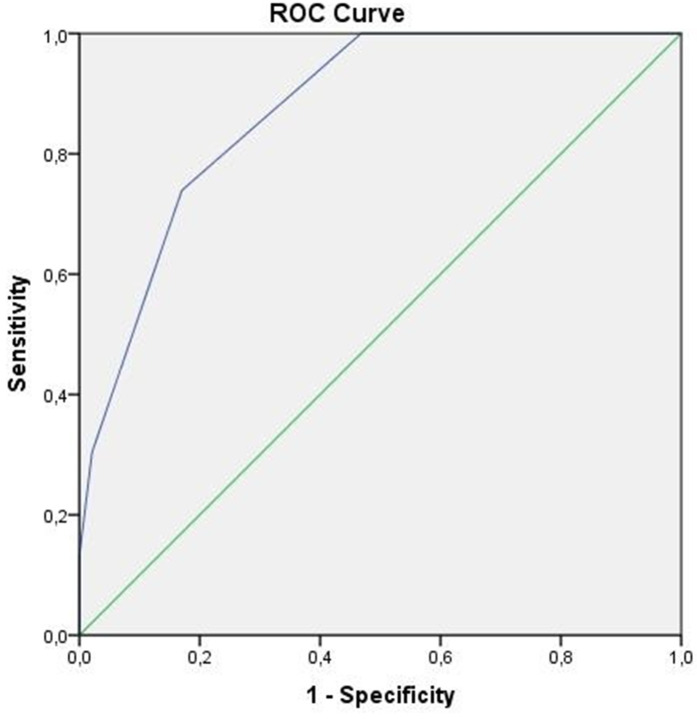
Receiver Operating Characteristic (ROC) analysis for optimal PAWSS cutoff.

In our sample, the ROC analysis showed that PAWSS ≥ 6 is the optimal cutoff point. The Area Under the Curve (AUC) was 0.873 which indicates a good diagnostic performance for the scale (Fig. 1). The sensitivity, specificity, positive and negative predictive values (PPV, NPV) and Youden’s Index for the different cutoff values are shown in Table 1. These results indicate that a PAWSS score of ≥ 6 has the highest predictive value for current c-AWS. Positive and negative predictive values and the occurrence of false positive and false negative diagnoses for the threshold value of 6 are in Table 2.

**Table 1:** Sensitivity, specificity, PPV and NPV report for ROC analysis.

Cutoff	Sensitivity (%)	Specificity (%)	PPV (%)	NPV(%)	Youden Index
4	100%	31.91%	41.82%	100%	0.319
5	100%	53.19%	51.11%	100%	0.531
**6**	**73.91%**	**82. 98%**	**68%**	**86.67%**	**0.568**

Note: The optimally efficient cutoff point is indicated in bold which is based on the sensitivity, specificity, PPV, NPV and Youden index measures. Abbreviations: PPV = Positive Predictive Value; NPV = Negative Predictive Value; ROC = Receiver Operating Characteristic

**Table 2:** Sensitivity, specificity, PPV and NPV calculations for the PAWSS cutoff of 6.

	c-AWS + N	c-AWS – N	TOTAL N
PAWSS +	True positives (TP) 17	False positives (FP) 8	All PAWSS + 25
PAWSS -	False negatives (FN) 6	True negatives (TN) 39	All PAWSS – 45
TOTAL	Patients with c-AWS (c-AWS+) 23	Total patients with no c-AWS (c-AWS-) 47	Total patients 70

Abbreviations: c-AWS = complicated alcohol withdrawal syndrome; AWS ‘+’ = presence of complicated alcohol withdrawal syndrome; AWS ‘−’ = no presence of complicated alcohol withdrawal syndrome; PAWSS = Prediction of Alcohol Withdrawal Severity Scale; PAWSS ‘+’ = PAWSS score of ≥6; PAWSS ‘−’ = PAWSS score of <6; TP = True Positive; FP = False positive; TN = True Negative; FN = False Negative; N = number of subjects per group.

To reveal the scale’s reliability, inter-rater reliability was measured by the Cohen’s Kappa coefficient. Based on the article by Maldonado and his colleagues [4], the PAWSS scores were treated as binary variables. In the first case, a PAWSS score of ≥6 indicated high risk for c-AWS, in the second case a PAWSS score of <6 did not indicate high risk for c-AWS. According to our results the Cohen’s Kappa coefficient was proven to be 1, which indicates perfect agreement between the two raters. It implies 45 cases of agreement on classifying patients as low-risk, 25 cases of agreement on classifying patients in the high-risk group and there were no cases of disagreement between the two evaluators.

In the case of the predictive validity of the PAWSS scale, the reference group was the ‘no c-AWS’ group. Results indicate that the PAWSS score of ≥6 was identified as a significant predictive factor for current c-AWS (OR = 12.332; 95% CI = 3.468–43.85; p < 0.001), which was independent from age, sex and the SADQ total score (Table 3).

**Table 3:** Binary logistic regression of the current c-AWS.

	MODEL 1		
	B (SE)	OR	95%CI
Age	−0.016 (0.032)	0.983	0.922-1.05
Sex	−0.495 (0.749)	0.609	0.140-2.64
SADQ total score	0.026 (0.024)	1.027	0.978- 1.08
PAWSS +	2.512 (0.647)	12.332***	**3.468-43.85**

Note: The reference group for the Models was the ‘no complicated AWS’ group. PAWSS = Prediction of Alcohol Withdrawal Severity Scale; PAWSS ‘+’ = PAWSS score of ≥6; B = unstandardized regression coefficient; CI = confidence interval; OR = odds ratio; SE = standard error.

(***p < 0.001)

### Revealing the role of the kindling mechanism in the development of c-AWS

The chi-square test results showed significant difference between the rate of the history of c-AWS and current c-AWS (χ2 (1) = 13.0; *p* < 0.001) and 21.4% (n = 15) of the patients had both current and previous c-AWS (Table 4). The Phi-coefficient was 0.431, which indicates that the history of c-AWS has a relatively strong effect on current c-AWS.

**Table 4:** Binary logistic regression of current c-AWS.

	MODEL 2		
	B (SE)	OR	95%CI
Age	−0.018 (0.028)	0.982	0.928-1.04
Sex	0.016 (0.670)	1.016	0.273-3.78
History of c-AWS	1.945 (0.578)	6.997***	**2.251-21.75**

Note: The reference group for the Models was the ‘no complicated AWS’ group. PAWSS = Prediction of Alcohol Withdrawal Severity Scale; AWS = alcohol withdrawal syndrome, B = unstandardized regression coefficient; CI = confidence interval; OR = odds ratio; SE = standard error.

(***p < 0.001)

Furthermore, a binary logistic regression was performed to examine the history of the c-AWS as a predictive variable of current c-AWS to explore the role of the kindling mechanism in the development of c-AWS. The reference group was the ‘no c-AWS group’. Based on the results, the history of the c-AWS (OR = 6.997; 95% CI = 2.251–21.75; p < 0.001) was a significant predictive factor for current c-AWS, when controlling for the demographic (age, sex) variables (Table 5).

**Table 5:** Chi-square test for the rate of the history of c-AWS and the current c-AWS.

	History of c-AWS			
Current c-AWS	No	Yes	*X* ^2^	Φ
No	37 52.9%	10 14.3%	13.0***	0.431
Yes	8 11.4%	15 21.4%		

Note: c-AWS = complicated alcohol withdrawal syndrome; *X*^2 ^= Chi-square; Φ = Phi-coefficient.

(***p < 0.001)

## Discussion

The complicated form of withdrawal states (c-AWS) can lead to life-threatening outcomes. It has been reported that the mortality rate of DT after optimal treatment is approximately 5% [28]. Furthermore, the occurrence of withdrawal-related seizures can lead to the development of status epilepticus [29,30]. Therefore, risk analysis of the development of c-AWS is crucial in clinical practice to reduce the lethal complications of ADS. Hence, based on a literature search, Maldonando and his colleagues developed a new clinical scale for determining the risk of developing c-AWS [4,5]. The scale includes various items that focus on the current state of the patient as well as the data of the previous withdrawal states. Although earlier studies have suggested that the kindling mechanism is involved in the development of DT and/or ARS [12,16,17,19], there is not enough clinical data on it. Our previous findings revealed that history of ARS and current DT are risk factors for developing ARS [16].

In this study, we first validated the Hungarian version of the PAWSS. To the best of our knowledge, the PAWSS has never been translated to any other language, and this was the first validation study of the questionnaire specifically among patients with ADS. Based on our results, the optimal cut-off score with the highest sensitivity (73.91%) and specificity (82.98%) is 6, which is different from the original validation study [4]. Maldonado and his colleagues reported that the AUC of the ROC analysis was 0.9765 [4]. These findings are in agreement with our results, which showed a similarly high diagnostic performance for the scale (AUC = 0.873). Regarding the scale’s reliability, the Cohen’s Kappa coefficient was 1, which indicates perfect agreement between the two evaluators in terms of inter-rater reliability statistics. This outcome supports the results of the original article [4] and confirms the reliability of the PAWSS. Moreover, in our sample the chance of the current c-AWS was 12 times higher among patients with PAWSS scores of 6 or higher, which indicates the high predictive validity of the scale. Thus, the present study demonstrated that the Hungarian version of the PAWSS is a reliable and valid tool for predicting c-AWS.

Particular attention should be paid to the fact that in the original validation study [4], the sample consisted of medically ill patients who were hospitalized in the general internal medicine and surgery wards. In this study, inpatients diagnosed with ADS (F10.20.) and the principal diagnosis of alcohol withdrawal state (F10.30) were examined. The enrolled patients were not critically ill and had no clinically significant neurological or somatic disorders. Because of the primary diagnosis of AUD, the patients probably had different drinking patterns and more serious alcohol dependence. This may be the reason for the higher cut-off score (≥ 6) in our study compared to the original validation of the PAWSS (cut-off ≥ 4).

Previous studies have recommended the use of the AUDIT or CAGE to screen high-risk patients for developing AWS [31]. However, Brousse and his colleagues proposed different thresholds for the AUDIT and CAGE for better predictive ability [32], and Ungur and his colleagues stated that by using the AUDIT, it is only possible to predict the risk and severity of AWS in half of AUD patients [33]. However, Dolman and Hawkes suggested that AUDIT alone is not satisfactory for detecting the risk of AWS [34]. In our study, the severity of alcohol dependence was measured using the SADQ, which is a more complex questionnaire that was developed to properly estimate the severity of dependence and help establish a more precise diagnosis [20].

In one of our two binary logistic regression models, the SADQ total score was not found to be a significant predictive factor for the current c-AWS. Thus, our results suggest that there might be little connection between the severity of dependence and the occurrence of c-AWS. In addition, it has been demonstrated that the development of c-AWS is independent of the severity of AWS [16]. Based on previous genetic research, it is assumed that patients who develop c-AWS are a specific subgroup of individuals with ADS [35–38].

Based on the second binary logistic regression model, a history of c-AWS was a significant predictor of current c-AWS, since the probability of current c-AWS was almost 7 times higher in the case of the history of c-AWS. This supports the role of kindling mechanism in the development of c-AWS. Our results highlight the need to introduce PAWSS in clinical settings. Furthermore, recognition of the high-risk subgroup of patients with c-AWS is essential, regarding the lethal consequences of it, e.g., status epilepticus or malignant arrythmias. In addition, preventive interventions are possible with the use of the PAWSS, such as the use of aggressive benzodiazepine therapy and supplementation of treatment with antiepileptic medications [3,4,16,39].

## Limitations

The first limitation of the study is that the present research was conducted with a small sample size, and the data collection took place at a single regional hospital in Hungary; therefore, the generalizability of our results is limited. Second, AWS scores were not assessed; however, it is essential to mention that the patients met the inclusion criteria of having an established ICD-10 diagnosis of the current AWS by a consultant psychiatrist. Finally, the history of c-AWS was detected using only one data point: yes or no. In addition to these limitations, it is important to note that the PAWSS has not yet been translated into other languages, its psychometric characteristics have not yet been determined in other countries and among patients with the principal diagnosis of ADS, and the kindling mechanism has not yet been tested using this scale.

## Conclusions

In conclusion, the present study validated the Hungarian version of the PAWSS, which has distinguished psychometric properties and a high predictive value for c-AWS among patients with ADS. Furthermore, our results revealed a history of c-AWS as a risk factor for the development of current complicated withdrawal. This suggests the importance of the kindling mechanism underlying the development of c-AWS. Additionally, our observations may suggest that patients who are suffering from c-AWS during withdrawal state comprise a distinct subpopulation of individuals diagnosed with ADS.

## Supporting information

S1 File The original version of the Prediction of Alcohol Withdrawal Severity Scale. (DOCX)

S2 File The Hungarian version of the Prediction of Alcohol Withdrawal Severity Scale. (DOCX)
