# Supplementary figures and images for: The role of kindling mechanism: A validation study of the Hungarian version of the Prediction of Alcohol Withdrawal Severity Scale
Source: PLoS One. 2025 Sep 2;20(9):e0330629. doi: 10.1371/journal.pone.0330629 (PMC12404368; doi:10.1371/journal.pone.0330629)

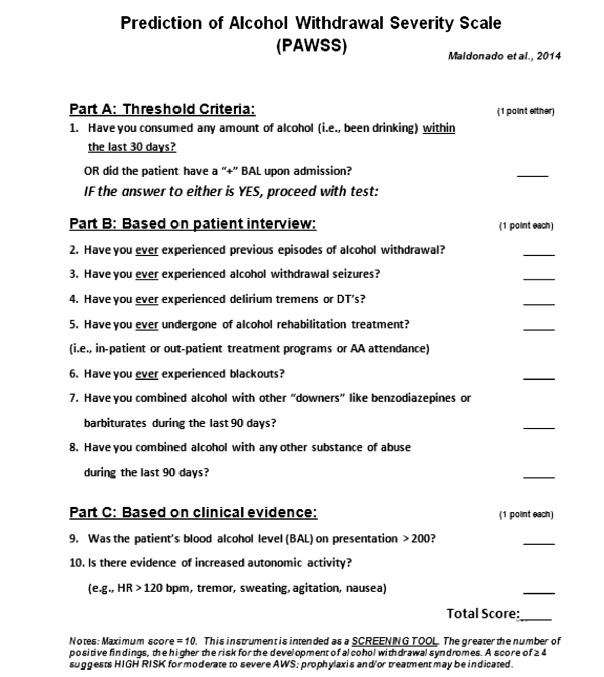
**The original version of the Prediction of Alcohol Withdrawal Severity Scale.**

Supplement: S1 File — (DOCX) [file pone.0330629.s001.docx]

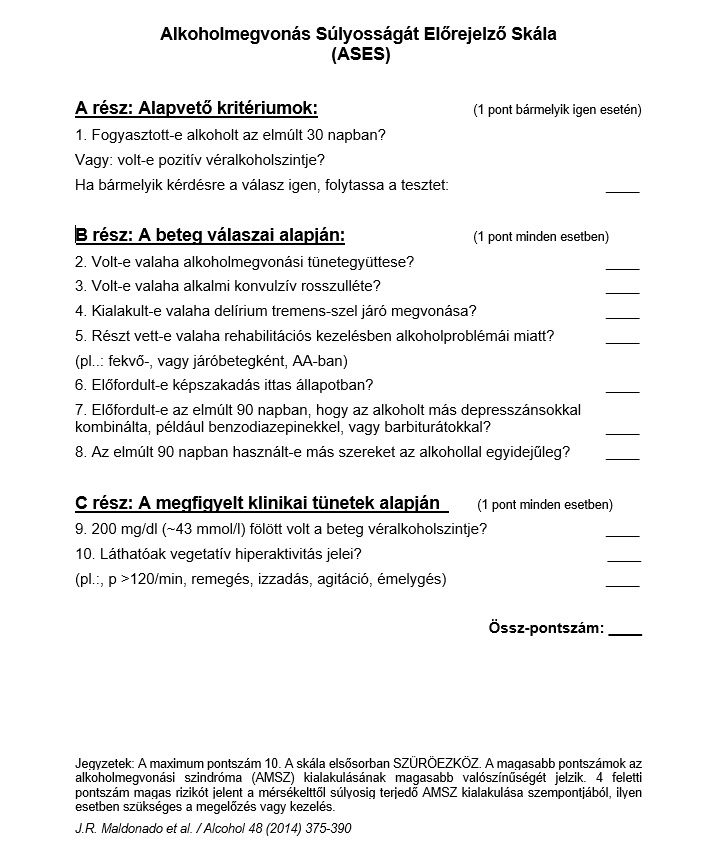
**The Hungarian version of the Prediction of Alcohol Withdrawal Severity Scale.**

Supplement: S2 File — (DOCX) [file pone.0330629.s002.docx]
